# Theoretical investigations on radiation generation of TEM, linearly or circularly polarized TE_n1_ coaxial waveguide mode in relativistic magnetron

**DOI:** 10.1038/s41598-017-01583-w

**Published:** 2017-05-04

**Authors:** Di-Fu Shi, Bao-Liang Qian, Hong-Gang Wang, Wei Li, Guang-Xing Du

**Affiliations:** College of Optoelectric Science and Engineering, National University of Defense Technology, Changsha, Hunan 410073 People’s Republic of China

## Abstract

The physical mechanism of the radiation generation of all possible output modes of the relativistic magnetron (RM) with all cavity-magnetron axial extraction technique is theoretically analysed, and the necessary conditions for generating these modes are obtained respectively. Assuming that *n*_0_ is the number of the electron spokes, *N* ≥ 4 as the total number of the cavities is an even number, and *k* is a nonnegative integer, some conclusions can be drawn as follows. If *n*_0_ = *kN* is true, no mode can be excited in the coaxial waveguide; if *n*_0_ = (2*k* + 1)*N*/4 is true, the linearly polarized modes can be excited in the coaxial waveguide; if *n*_0_ = (4*k* + 2)*N*/4 is true, the TEM mode and the linearly polarized modes can be excited in the coaxial waveguide; if *n*_0_ takes other value, the left and right circularly polarized modes can be excited in the coaxial waveguide and the directions of rotation of the circularly polarized modes can be reversed with the reversion of the direction of rotation of the electron spokes; in addition, some other regular characteristics of the corresponding mode excitation are presented in detail in this paper. Such unique attractive properties that have been verified by the cold and hot simulations in this paper make it possible for this type of RM to meet application requirements of various high power microwave (HPM) modes.

## Introduction

Compared with linearly polarized microwaves, circularly polarized microwaves have been widely used in radar, navigation, guidance, communications, television broadcasts systems and so on, because most antennas that have different polarization directions can always receive circularly polarized microwaves. At present, high power microwave (HPM) sources of various types of structure and principle can generate microwaves with different modes in the output waveguide. However, except for the relativistic magnetron (RM), HPM sources that can generate circularly polarized microwaves without using mode converters are rarely reported. In fact, RMs can generate separately various microwave modes, such as a TE_01_ mode^1, 2^, a linearly polarized TE_n1_ (n ≥ 1 is an integer) mode^1–5^, or a circularly polarized TE_n1_ (n ≥ 2 is an integer) mode^4–7^ in circular waveguide, or a TE_10_ mode in rectangular waveguide^8, 9^. Until 2012, RMs with all cavity-magnetron axial extraction technique were investigated with numerical simulation^10^ and it has become possible to generate TEM modes in a coaxial waveguide. Then based on this technique, in 2016 a compact RM with the circularly polarized TE_11_ mode in a coaxial waveguide was proposed and verified by theoretical and simulation investigations^11^. And due to the uniqueness of the output mode, the RM seems more attractive and competitive than other HPM sources. However, comprehensively and thoroughly theoretical investigations on the radiation generation of all possible output modes of the RM with all cavity-magnetron axial extraction technique have not yet appeared in relevant literature.

In this paper, based on vector Helmholtz equation and field matching method, we have systematically analysed the physical mechanism of the radiation generation of all possible output modes of the RM with all cavity-magnetron axial extraction technique and have respectively obtained the necessary conditions for generating TEM mode, linearly polarized TE_n1_ mode, and circularly polarized TE_n1_ mode in coaxial waveguide of the RM. Furthermore, the correctness of the theoretical analysis has been verified by the cold and hot simulations.

## Theoretical analysis of mode excitation

**Figure 1:**
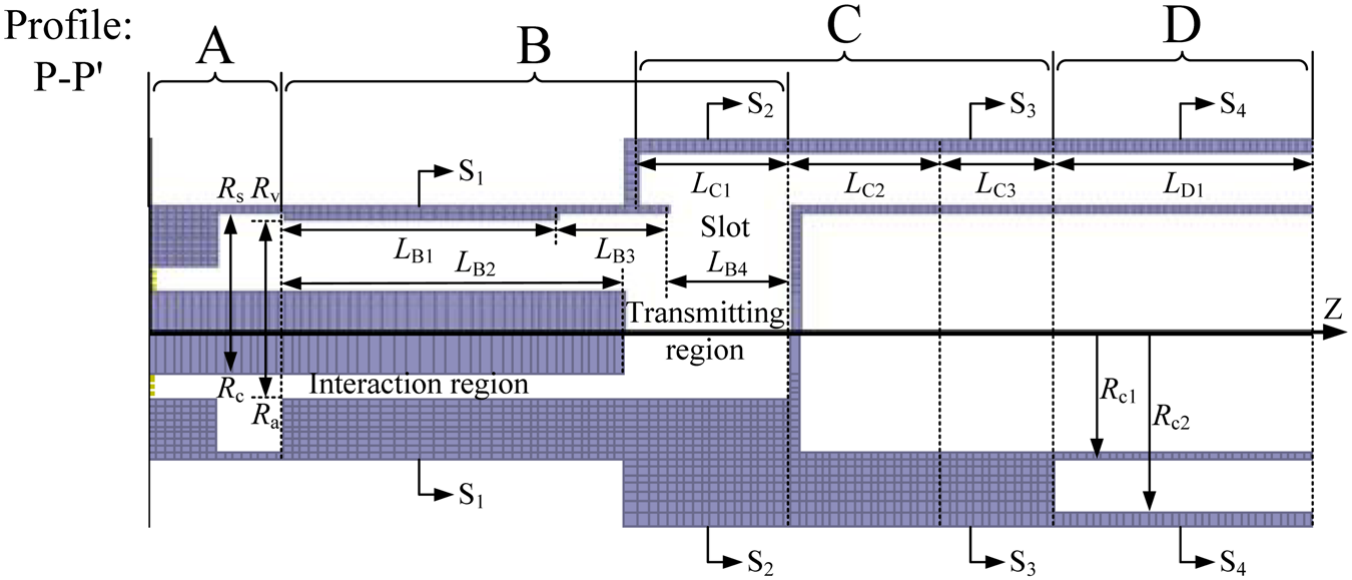
Schematic diagram of the longitudinal profile of the RM.

**Figure 2:**
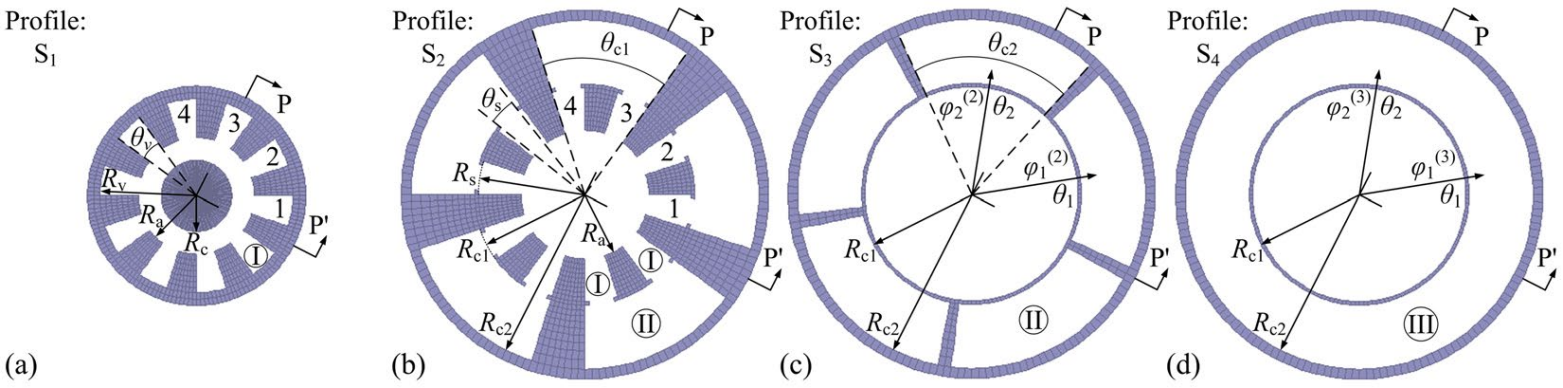
Schematic diagram of the transversal profiles of the RM in the (**a**) S_1_ plane, (**b**) S_2_ plane, (**c**) S_3_ plane, and (**d**) S_4_ plane.

Without loss of generality, a ten-cavity RM with all cavity-magnetron axial extraction technique, as was discussed in ref. 11, is taken into consideration. The longitudinal profile of the RM and the transversal profiles of the RM in the planes of S_1_, S_2_, S_3_, and S_4_ are shown in Figs 1 and 2 respectively. The whole structure of the RM is divided into four sections, namely section A of the coaxial waveguide for electric power input, section B of the resonant system for beam-wave interaction, section C of the sectorial waveguides arranged in an annulus for microwave extraction, and section D of the coaxial waveguide for microwave output. In the model, a resonant system with ten sector-type cavities is adopted. In the axial direction, *L*_B1_ indicates the length of the beam-wave interaction region; *L*_B2_ indicates the length of the cathode; *L*_B4_ indicates the length of the coupling slots; *L*_B3_ indicates the distance between the interaction region and the coupling slots; *L*_C1_ indicates the length of the anterior segment of the sectorial waveguides; *L*_C2_ indicates the length of the transition segment of the sectorial waveguides; *L*_C3_ indicates the length of the posterior segment of the sectorial waveguides; *L*_D1_ indicates the length of the coaxial waveguide in section D. In the transverse direction, *R*_c_ indicates the cathode radius; *R*_a_ indicates the anode radius; *R*_v_ indicates the cavity radius in the interaction region; *R*_s_ indicates the cavity radius in the transmitting region; *R*_c1_ indicates the inner radius of the sectorial waveguides; *R*_c2_ indicates the outer radius of the sectorial waveguides; *θ*_v_ indicates the cavity angle; *θ*_s_ indicates the coupling slot angle; *θ*_c1_ indicates the angle of the anterior segment of the sectorial waveguides; *θ*_c2_ indicates the angle of the posterior segment of the sectorial waveguides.

For the sake of convenient narration in the theoretical analysis, the cylindrical-coordinate system is established at the center of the RM; the observation in this paper is along the -Z direction; the cavities in section B are respectively numbered *p* = 1, ……, *N* in a counterclockwise direction, where the total number of the cavities *N* is an even number equal to or greater than 4; the area in which RF fields exist is divided into three parts: area I of the cavities (*R*_*a*_ < *r* < *R*_*s*_) with the longitudinal range of *L*_B1_ + *L*_B3_ + *L*_B4_ in section B, area II of the sectorial waveguide (*R*_*c*1_ < *r* < *R*_*c*2_) with the longitudinal range of *L*_C1_ + *L*_C2_ + *L*_C3_ in section C, and area III of the coaxial waveguide (*R*_*c*1_ < *r* < *R*_*c*2_) with the longitudinal range of *L*_D1_ in section D. In addition, all the theoretical analysis below is based on the premise that only the TE_11_ mode can propagate steadily in the sectorial waveguides of section C. Under the premise, the reflected waves with other waveguide modes in section C can not be excited yet. Thus, the reflected waves can not affect the output mode components in the coaxial waveguide of section D, and only the waves in the +Z direction are discussed here.

### Analysis for area I

In area I, as the cavities with sectorial type are adopted, the axial component of magnetic field *H*_*z*_^(1)^(*r*, *θ*, *z*) in a sectorial cavity can be written as

1 $${H}_{z}^{(1)}(r,\theta ,z)={H}_{z0}^{(1)}[{N}_{{v}_{1}}^{\prime} ({k}_{c1}{R}_{s}){J}_{{v}_{1}}({k}_{c1}r)-{J}_{{v}_{1}}^{\prime} ({k}_{c1}{R}_{s}){N}_{{v}_{1}}({k}_{c1}r)]\cos ({v}_{1}\theta ){e}^{-j{\beta }_{1}z},$$

where *H*_*z*0_^(1)^ is a constant, *v*_1_ = *n*_1_π/*θ*_v_, *n*_1_ as the azimuthal mode number is a positive integer, *J*_*v*1_(*k*_*c*1_ *r*) and *N*_*v*1_(*k*_c1_ *r*) are the well-known Bessel and Neumann functions of order *v*_1_ respectively, *k*_*c*1_ is the transverse cutoff wave number, and *β*_1_ is the axial propagation constant. A previous study has shown that the lowest azimuthal mode in the cavities dominants the beam-wave interaction of a RM^12^, therefore *n*_1_ = 0 and with Eq. (1) the azimuthal component of electric field in a sectorial cavity can be obtained as

2 $${E}_{\theta }^{(1)}(r,\theta ,z)=\frac{j\omega \mu }{{k}_{c1}}\frac{{\rm{\partial }}{H}_{z}^{(1)}(r,\theta ,z)}{{\rm{\partial }}r}={E}_{\theta }^{(1)}(r){e}^{-j{\beta }_{1}z},$$

where *E*_*θ*_^(1)^(*r*) can be given as

3 $${E}_{\theta }^{(1)}(r)=\frac{j\omega \mu }{{k}_{c1}}{H}_{z0}^{(1)}[{N}_{0}^{\prime} ({k}_{c1}{R}_{s}){J}_{0}^{\prime} ({k}_{c1}r)-{J}_{0}^{\prime} ({k}_{c1}{R}_{s}){N}_{0}^{\prime} ({k}_{c1}r)].$$

Due to the periodically azimuthal symmetry of the cavities, the electromagnetic fields in the cavities have the same amplitude but with different phases. The phase difference between two adjacent cavities is 2π*n*_0_/*N*, where *n*_0_ representing the number of the electron spokes indicates the azimuthal periodicity of the electromagnetic field distribution. Thus, with Eq. (2) the azimuthal component of electric field in a cavity can be expressed as

4 $${E}_{\theta ,p}^{(1)}(r,\theta ,z)={E}_{\theta }^{(1)}(r,\theta ,z){e}^{\pm j2\pi {n}_{0}p/N},$$

where “−” and “+” indicate the electron spokes rotate counterclockwise and clockwise respectively.

Without loss of generality the azimuthal components of the electric fields in the first four adjacent cavities which correspond to the first two adjacent sectorial waveguides are taken into consideration and with Eq. (4) they can be respectively given as

5 $$\{\begin{array}{l}{E}_{\theta ,1}^{(1)}(r,\theta ,z)={E}_{\theta }^{(1)}(r,\theta ,z){e}^{\pm j2\pi {n}_{0}\cdot 1/N},\\ {E}_{\theta ,2}^{(1)}(r,\theta ,z)={E}_{\theta }^{(1)}(r,\theta ,z){e}^{\pm j2\pi {n}_{0}\cdot 2/N},\\ {E}_{\theta ,3}^{(1)}(r,\theta ,z)={E}_{\theta }^{(1)}(r,\theta ,z){e}^{\pm j2\pi {n}_{0}\cdot 3/N},\\ {E}_{\theta ,4}^{(1)}(r,\theta ,z)={E}_{\theta }^{(1)}(r,\theta ,z){e}^{\pm j2\pi {n}_{0}\cdot 4/N}.\end{array}$$

### Analysis for area II

In area II, the axial component of magnetic field *H*_*z*_^(2)^(*r*, *θ*, *z*) in a sectorial waveguide can be written as

6 $${H}_{z}^{(2)}(r,\theta ,z)={H}_{z0}^{(2)}[{N}_{{v}_{2}}^{\prime} ({k}_{c2}{R}_{c2}){J}_{{v}_{2}}({k}_{c2}r)-{J}_{{v}_{2}}^{\prime} ({k}_{c2}{R}_{c2}){N}_{{v}_{2}}({k}_{c2}r)]\cos ({v}_{2}\theta ){e}^{-j{\beta }_{2}z},$$

where *H*_*z*0_^(2)^ is a constant, *v*_2_ = *n*_2_π/*θ*_c2_, *n*_2_ as the azimuthal mode number is a positive integer, *J*_*v*2_(*k*_c2_ *r*) and *N*_*v*2_(*k*_c2_ *r*) are the well-known Bessel and Neumann functions of order *v*_2_ respectively, *k*_*c*2_ is the transverse cutoff wave number, and *β*_2_ is the axial propagation constant. Since only the TE_11_ mode can propagate steadily in the sectorial waveguides, *n*_2_ = 1 and with Eq. (6) the radial component of electric field in a sectorial waveguide can be obtained as

7 $${E}_{r}^{(2)}(r,\theta ,z)=-\,\frac{j\omega \mu }{{k}_{c2}^{2}r}\frac{\partial {H}_{z}^{(2)}(r,\theta ,z)}{\partial \theta }={E}_{r}^{(2)}(r)\sin ({v}_{2}\theta ){e}^{-j{\beta }_{2}z},$$

where *E*_*r*_^(2)^(r) can be given as

8 $${E}_{r}^{(2)}(r)=\frac{j\omega \mu {v}_{2}}{{k}_{c2}^{2}r}{H}_{z0}^{(2)}[{N}_{{v}_{2}}^{\prime} ({k}_{c2}{R}_{c2}){J}_{{v}_{2}}({k}_{c2}r)-{J}_{{v}_{2}}^{\prime} ({k}_{c2}{R}_{c2}){N}_{{v}_{2}}({k}_{c2}r)].$$

Due to the bilateral symmetry between the extraction structures of the odd and even cavities in section C, all the amplitudes of the electric fields can vary from *E*_*θ*_^(1)^(*r*, *θ*, *z*) in the cavities to *E*_*r*_^(2)^(*r*, *θ*, *z*) in the sectorial waveguides with the same variation, while the corresponding phase variations of the electric fields have some difference. Assuming that the phase variations of the electric fields from the odd cavities are e^*jα*^, the phase variations of the electric fields from the even cavities would be e^*jα*^e^*jπ*^. And with Eqs (5) and (7) the radial components of the electric fields in the first two sectorial waveguides extracted from the first four adjacent cavities can be respectively obtained as

9 $$\{\begin{array}{l}{E}_{r,1}^{(2)}(r,{\theta }_{1},z)={E}_{r}^{(2)}(r,{\theta }_{1},z){e}^{\pm j2\pi {n}_{0}\cdot 1/N}{e}^{j\alpha },\\ {E}_{r,2}^{(2)}(r,{\theta }_{1},z)={E}_{r}^{(2)}(r,{\theta }_{1},z){e}^{\pm j2\pi {n}_{0}\cdot 2/N}{e}^{j\alpha }{e}^{j\pi },\\ {E}_{r,3}^{(2)}(r,{\theta }_{2},z)={E}_{r}^{(2)}(r,{\theta }_{2},z){e}^{\pm j2\pi {n}_{0}\cdot 3/N}{e}^{j\alpha },\\ {E}_{r,4}^{(2)}(r,{\theta }_{2},z)={E}_{r}^{(2)}(r,{\theta }_{2},z){e}^{\pm j2\pi {n}_{0}\cdot 4/N}{e}^{j\alpha }{e}^{j\pi },\end{array}$$

where *θ*_1_ and *θ*_2_ are the angular coordinates of the first two sectorial waveguides respectively, corresponding to the phases of *φ*_1_^(2)^ and *φ*_2_^(2)^.

For *n*_0_ = *kN*, where *k* is a nonnegative integer, with Eq. (9) the radial components of the total electric fields in the first two sectorial waveguides are both equal to zero as shown in Eq. (10), as well as in other sectorial waveguides of section C.

10 $${E}_{r,1}^{(2)}(r,{\theta }_{1},z)+{E}_{r,2}^{(2)}(r,{\theta }_{1},z)={E}_{r,3}^{(2)}(r,{\theta }_{2},z)+{E}_{r,4}^{(2)}(r,{\theta }_{2},z)=0.$$

Therefore, no waveguide mode will be excited in the sectorial waveguides, neither in the coaxial waveguide of section D.

For *n*_0_ ≠ *kN*, with Eq. (9) the phase difference ∆*φ* ^(2)^ = *φ*_2_^(2)^ − *φ*_1_^(2)^ between the radial components of the total electric fields in the first two sectorial waveguides can be obtained as shown in Eq. (11), as well as in other two adjacent sectorial waveguides of section C.

11 $$\frac{{E}_{r,3}^{(2)}(r,{\theta }_{2},z)+{E}_{r,4}^{(2)}(r,{\theta }_{2},z)}{{E}_{r,1}^{(2)}(r,{\theta }_{1},z)+{E}_{r,2}^{(2)}(r,{\theta }_{1},z)}={e}^{\pm j4\pi {n}_{0}/N}={e}^{j{\rm{\Delta }}{\phi }^{(2)}}\cdot $$

### Analysis for area III

In area III, for TEM mode, the radial component of electric field in the coaxial waveguide can be written as

12 $${E}_{r,{\rm{TEM}}}^{(3)}(r,z)={E}_{r,{\rm{TEM}}}^{(3)}(r){e}^{-j{k}_{{\rm{TEM}}}z},$$

where *E*_r, TEM_^(3)^(*r*) = *E*_0_/*r*, *E*_0_ is a constant, and *k*_TEM_ is the transverse cutoff wave number.

For TE_n1_ mode, assuming that the axial component of magnetic field *H*_*z*, TE_^(3)^(*r*, *θ*, *z*) in the coaxial waveguide is an even function about *θ* = 0 without loss of generality, one can get

13 $$\begin{array}{l}{H}_{z,{\rm{TE}}}^{(3)}(r,\theta ,z)={H}_{z0,{n}_{3}}^{(3)}[{N}_{{n}_{3}}^{\prime} ({k}_{c3,{n}_{3}}{R}_{c2}){J}_{{n}_{3}}({k}_{c3,{n}_{3}}r)-{J}_{{n}_{3}}^{\prime} ({k}_{c3,{n}_{3}}{R}_{c2}){N}_{{n}_{3}}({k}_{c3,{n}_{3}}r)]\cos ({n}_{3}\theta ){e}^{-j{\beta }_{3,{n}_{3}}z},\end{array}$$

where *H*_*z*0,*n*3_^(3)^ is a constant, *n*_3_ as the azimuthal mode number is a positive integer, *J*_*n*3_(*k*_c3,*n*3_ *r*) and *N*_*n*3_(*k*_c3,*n*3_ *r*) are the well-known Bessel and Neumann functions of order *n*_3_ respectively, *k*_*c*3,*n*3_ is the transverse cutoff wave number, and *β*_3,*n*3_ is the axial propagation constant. With Eq. (13) the radial component of electric field in the coaxial waveguide can be obtained as

14 $${E}_{r,{\rm{TE}}}^{(3)}(r,\theta ,z)=-\,\frac{j\omega \mu }{{k}_{c3,{n}_{3}}^{2}r}\frac{\partial {H}_{z,{\rm{TE}}}^{(3)}(r,\theta ,z)}{\partial \theta }={E}_{r,{\rm{TE}}}^{(3)}(r)\sin ({n}_{3}\theta ){e}^{-j{\beta }_{3,{n}_{3}}z},$$

where *E*_*r*, TE_^(3)^(*r*) can be given as

15 $${E}_{r,{\rm{TE}}}^{(3)}(r)=\frac{j\omega \mu {n}_{3}}{{k}_{c3,{n}_{3}}^{2}r}{H}_{z0,{n}_{3}}^{(3)}[{N}_{{n}_{3}}^{\prime} ({k}_{c3,{n}_{3}}{R}_{c2}){J}_{{n}_{3}}({k}_{c3,{n}_{3}}r)-{J}_{{n}_{3}}^{\prime} ({k}_{c3,{n}_{3}}{R}_{c2}){N}_{{n}_{3}}({k}_{c3,{n}_{3}}r)].$$

In order to construct an electromagnetic field function of a circularly polarized coaxial waveguide mode, the radial components of electric fields of two polarized degenerate modes with an angular phase difference of π/2 are required.

For left circular polarization, with Eq. (14) the radial components of electric fields of two polarized degenerate modes can be written as

16 $$\{\begin{array}{l}{E}_{r,{\rm{TE}},L1}^{(3)}(r,\theta ,z)={E}_{r,{\rm{TE}},L}^{(3)}(r)\sin ({n}_{L}\theta ){e}^{-j{\beta }_{3,{n}_{L}}z},\\ {E}_{r,{\rm{TE}},L2}^{(3)}(r,\theta ,z)={E}_{r,{\rm{TE}},L}^{(3)}(r)\sin ({n}_{L}\theta -\frac{\pi }{2}){e}^{-j\pi /2}{e}^{-j{\beta }_{3,{n}_{L}}z},\end{array}$$

where *n*_*L*_ is used instead of *n*_3_. With Eq. (16) the radial component of total electric field of the left circularly polarized coaxial waveguide mode can be obtained as

17 $${E}_{r,{\rm{TE}},L}^{(3)}(r,\theta ,z)={E}_{r,{\rm{TE}},L1}^{(3)}(r,\theta ,z)+{E}_{r,{\rm{TE}},L2}^{(3)}(r,\theta ,z)={E}_{r,{\rm{TE}},L}^{(3)}(r){e}^{j(\pi /2-{n}_{L}\theta )}{e}^{-j{\beta }_{3,{n}_{L}}z}.$$

For right circular polarization, with Eq. (14) the radial components of electric fields of two polarized degenerate modes can be written as

18 $$\{\begin{array}{l}{E}_{r,{\rm{TE}},R1}^{(3)}(r,\theta ,z)={E}_{r,{\rm{TE}},R}^{(3)}(r)\sin ({n}_{R}\theta ){e}^{-j{\beta }_{3,{n}_{R}}z},\\ {E}_{r,{\rm{TE}},R2}^{(3)}(r,\theta ,z)={E}_{r,{\rm{TE}},R}^{(3)}(r)\sin ({n}_{R}\theta -\frac{\pi }{2}){e}^{j\pi /2}{e}^{-j{\beta }_{3,{n}_{R}}z},\end{array}$$

where *n*_*R*_ is used instead of *n*_3_. With Eq. (18) the radial component of total electric field of the right circularly polarized coaxial waveguide mode can be obtained as

19 $${E}_{r,{\rm{TE}},R}^{(3)}(r,\theta ,z)={E}_{r,{\rm{TE}},R1}^{(3)}(r,\theta ,z)+{E}_{r,{\rm{TE}},R2}^{(3)}(r,\theta ,z)={E}_{r,{\rm{TE}},R}^{(3)}(r){e}^{j({n}_{R}\theta -\pi /2)}{e}^{-j{\beta }_{3,{n}_{R}}z}.$$

### Solutions for matched fields

The electric fields of area II and area III should be matched on the boundary of the two areas. With Eqs (12), (17) and (19) the phase difference Δ*φ* ^(3)^ = *φ*_2_^(3)^−*φ*_1_^(3)^ between the radial components of the total electric fields with the angle difference of Δ*θ* = *θ*_2_ − *θ*_1_ = 4π/*N* in the coaxial waveguide should be equal to the phase difference Δ*φ* ^(2)^ between the radial components of the total electric fields in the first two sectorial waveguides in Eq. (11), as shown in Eq. (20).

20 $$\frac{{E}_{r,{\rm{TEM}}}^{(3)}(r,z)+\sum _{{n}_{L}=1}^{\infty }{E}_{r,{\rm{TE}},L}^{(3)}(r,{\theta }_{2},z)+\sum _{{n}_{R}=1}^{\infty }{E}_{r,{\rm{TE}},R}^{(3)}(r,{\theta }_{2},z)}{{E}_{r,{\rm{TEM}}}^{(3)}(r,z)+\sum _{{n}_{L}=1}^{\infty }{E}_{r,{\rm{TE}},L}^{(3)}(r,{\theta }_{1},z)+\sum _{{n}_{R}=1}^{\infty }{E}_{r,{\rm{TE}},R}^{(3)}(r,{\theta }_{1},z)}={e}^{j{\rm{\Delta }}{\phi }^{(3)}}={e}^{j{\rm{\Delta }}{\phi }^{(2)}}={e}^{\pm j4\pi {n}_{0}/N},$$

where *θ*_1_ and *θ*_2_ are the angular coordinates of the first two sectorial waveguides respectively, corresponding to the phases of *φ*_1_^(3)^ and *φ*_2_^(3)^. Thus,

21 $$\begin{array}{c}{E}_{r,{\rm{TEM}}}^{(3)}(r)[1-{e}^{\pm j4\pi {n}_{0}/N}]{e}^{-j{k}_{{\rm{TEM}}}z}\\ +\sum _{{n}_{L}=1}^{\infty }{E}_{r,{\rm{TE}},L}^{(3)}(r)[{e}^{j(\pi /2-{n}_{L}{\theta }_{2})}-{e}^{j(\pi /2-{n}_{L}{\theta }_{1})\pm j4\pi {n}_{0}/N}]{e}^{-j{\beta }_{3,{n}_{L}}z}\\ +\sum _{{n}_{R}=1}^{\infty }{E}_{r,{\rm{TE}},R}^{(3)}(r)[{e}^{j({n}_{R}{\theta }_{2}-\pi /2)}-{e}^{j({n}_{R}{\theta }_{1}-\pi /2)\pm j4\pi {n}_{0}/N}]{e}^{-j{\beta }_{3,{n}_{R}}z}=0.\end{array}$$

Taking *n*_0_ ≠ *kN* and general phase difference into consideration, the solutions of Eq. (21) can be described as follows.

For the counterclockwise rotation of electron spokes where “±” takes the minus, the solutions of generating the TEM mode, the left and right circularly polarized TE_n1_ coaxial waveguide modes can be respectively obtained as

22 $$\{\begin{array}{l}{n}_{0}=\frac{N}{2}(2k+1),\,\,\,k\in Z\,{\rm{and}}\,k\ge 0,\\ {n}_{L}={n}_{0}-\frac{N}{2}{p}_{L},\,\,{p}_{L}\in Z\,{\rm{and}}\,{p}_{L} < \frac{2{n}_{0}}{N},\\ {n}_{R}=-\,{n}_{0}+\frac{N}{2}{p}_{R},\,\,\,{p}_{R}\in Z\,{\rm{and}}\,{p}_{R} > \frac{2{n}_{0}}{N}.\end{array}$$

where Z is the set of all integers.

For the clockwise rotation of electron spokes where “±” takes the plus, the solutions of generating the TEM mode, the left and right circularly polarized TE_n1_ coaxial waveguide modes can be respectively obtained as

23 $$\{\begin{array}{l}{n}_{0}=\frac{N}{2}(2k+1),\,\,\,k\in Z\,{\rm{and}}\,k\ge 0,\\ {n}_{L}=-\,{n}_{0}-\frac{N}{2}{q}_{L},\,\,{q}_{L}\in Z\,{\rm{and}}\,{q}_{L} < -\,\frac{2{n}_{0}}{N},\\ {n}_{R}={n}_{0}+\frac{N}{2}{q}_{R},\,\,\,{q}_{R}\in Z\,{\rm{and}}\,{q}_{R} > -\,\frac{2{n}_{0}}{N}.\end{array}$$

where Z is the set of all integers.

According to Eqs (22) and (23), the necessary condition for *n*_*L*_ = *n*_*R*_ is *n*_0_ = (2*k* + 1)*N*/4 or *n*_0_ = (4*k* + 2)*N*/4 where *k* is a nonnegative integer, thus three cases have to be discussed as follows.

1. Firstly, if *n*_0_ = (2*k* + 1)*N*/4 is true, Eq. (11) can be written as
  24 $$\frac{{E}_{r,3}^{(2)}(r,{\theta }_{2},z)+{E}_{r,4}^{(2)}(r,{\theta }_{2},z)}{{E}_{r,1}^{(2)}(r,{\theta }_{1},z)+{E}_{r,2}^{(2)}(r,{\theta }_{1},z)}=-\,1.$$
  It means that the electric fields in any two adjacent sectorial waveguides have the same amplitude but with a phase difference of 180°.
  For the counterclockwise rotation of electron spokes, according to Eq. (22), the TEM mode can not be excited.
  For circularly polarized mode, since *n*_*L*_ and *n*_*R*_ from Eq. (22) can be rewritten as
  25 $$\{\begin{array}{c}{n}_{L}=\frac{N}{4}[(2k+1)-2{p}_{L}],\,\,{p}_{L}\in Z\,{\rm{and}}\,{p}_{L} < \frac{1}{2}(2k+1),\\ {n}_{R}=\frac{N}{4}[2{p}_{R}-(2k+1)],\,\,{p}_{R}\in Z\,{\rm{and}}\,{p}_{R} > \frac{1}{2}(2k+1),\end{array}$$
  they can be equivalent to the same arithmetic progression with the first term of *N*/4 and the common difference of *N*/2, as shown in Eq. (26).
  26 $${n}_{L}={n}_{R}=\frac{N}{4}+t\frac{N}{2},\,\,\,t\in Z\,{\rm{and}}\,t\ge 0.$$
  Thus, the left and right circularly polarized modes have the same set of azimuthal mode numbers of (*N*/4 + *tN*/2) but with different directions of rotation, as a result that the linearly polarized modes consisting of the corresponding left and right circularly polarized modes can be excited, and the solutions of generating the linearly polarized modes for different values of *n*_0_ are equivalent.
  For the clockwise rotation of electron spokes, the same conclusion also can be obtained.
2. Secondly, if *n*_0_ = (4*k* + 2)*N*/4 is true, Eq. (11) can be written as
  27 $$\frac{{E}_{r,3}^{(2)}(r,{\theta }_{2},z)+{E}_{r,4}^{(2)}(r,{\theta }_{2},z)}{{E}_{r,1}^{(2)}(r,{\theta }_{1},z)+{E}_{r,2}^{(2)}(r,{\theta }_{1},z)}=1$$
  It means that the electric fields in any two adjacent sectorial waveguides have the same amplitude and phase.
  For the counterclockwise rotation of electron spokes, according to Eq. (22), the TEM mode can be excited.
  For circularly polarized mode, since *n*_*L*_ and *n*_*R*_ from Eq. (22) can be rewritten as
  28 $$\{\begin{array}{c}{n}_{L}=\frac{N}{2}[(2k+1)-{p}_{L}],\,\,\,{p}_{L}\in Z\,{\rm{and}}\,{p}_{L} < 2k+1,\\ {n}_{R}=\frac{N}{2}[{p}_{R}-(2k+1)],\,\,\,{p}_{R}\in Z\,{\rm{and}}\,{p}_{R} > 2k+1,\end{array}$$
  they can be equivalent to the same arithmetic progression with the first term of *N*/2 and the common difference of *N*/2, as shown in Eq. (29).
  29 $${n}_{L}={n}_{R}=\frac{N}{2}+t\frac{N}{2},\,\,\,t\in Z\,{\rm{and}}\,t\ge 0.$$
  Thus, the left and right circularly polarized modes have the same set of azimuthal mode numbers of (*N*/2 + *tN*/2) but with different directions of rotation, as a result that the linearly polarized modes consisting of the corresponding left and right circularly polarized modes can be excited, and the solutions of generating the TEM mode and the linearly polarized modes for different values of *n*_0_ are equivalent.
  For the clockwise rotation of electron spokes, the same conclusion also can be obtained.
3. Thirdly, if *n*_0_ takes other values, *n*_*L*_ ≠ *n*_*R*_.

For the counterclockwise rotation of electron spokes, according to Eq. (22), the TEM mode can not be excited, while the left circularly polarized modes with the set of azimuthal mode numbers of (*n*_0_ − *p*_*L*_ *N*/2) and the right circularly polarized modes with the set of azimuthal mode numbers of (−*n*_0_ + *p*_*R*_ *N*/2) can be excited. Comparing the two cases of the numbers of the electron spokes of *n*_0_′ = *n*_0_ + *s*_1_ *N*/2 and *n*_0_ where *s*_1_ > −2*n*_0_/*N* is an integer, with Eq. (22) one can see that the solutions of generating the left and right circularly polarized modes for *n*_0_′ and *n*_0_ are equivalent. Comparing the two cases of the numbers of the electron spokes of *n*_0_′′ = −*n*_0_ + *s*_2_ *N*/2 and *n*_0_ where *s*_2_ > 2*n*_0_/*N* is an integer, with Eq. (22) one can see that the solutions of generating the left and right circularly polarized modes for *n*_0_′′ are equivalent to the solutions of generating the right and left circularly polarized modes for *n*_0_ respectively.

For the clockwise rotation of electron spokes, the similar conclusions also can be obtained.

In addition, comparing Eq. (22) with Eq. (23), one can see that the solutions of generating the left and right circularly polarized modes for the counterclockwise rotation of electron spokes are equivalent to the solutions of generating the right and left circularly polarized modes for the clockwise rotation of electron spokes respectively. It means that the directions of rotation of the circularly polarized modes can be reversed with the reversion of the direction of rotation of the electron spokes.

### Summary

To sum up briefly, at least four conclusions, where *k* and *t* are nonnegative integers, can be drawn as follows.

1. If *n*_0_ = *kN* is true, no mode can be excited in the coaxial waveguide.
2. If *n*_0_ = (2*k* + 1)*N*/4 is true, the linearly polarized modes with the set of azimuthal mode numbers of (*N*/4 + *tN*/2) can be excited in the coaxial waveguide, and the solutions of generating the linearly polarized modes for different values of *n*_0_ are equivalent.
3. If *n*_0_ = (4*k* + 2)*N*/4 is true, the TEM mode and the linearly polarized modes with the set of azimuthal mode numbers of (*N*/2 + *tN*/2) can be excited in the coaxial waveguide, and the solutions of generating the TEM mode and the linearly polarized modes for different values of *n*_0_ are equivalent.
4. If *n*_0_ takes other value, the left and right circularly polarized modes with their corresponding sets of azimuthal mode numbers can be excited in the coaxial waveguide. Comparing the two cases of the numbers of the electron spokes of *n*_0_′ = *n*_0_ + *s*_1_ *N*/2 and *n*_0_ where *s*_1_ > −2*n*_0_/*N* is an integer, the solutions of generating the left and right circularly polarized modes for *n*_0_′ and *n*_0_ are equivalent. Comparing the two cases of the numbers of the electron spokes of *n*_0_′′ = −*n*_0_ + *s*_2_ *N*/2 and *n*_0_ where *s*_2_ > 2*n*_0_/*N* is an integer, the solutions of generating the left and right circularly polarized modes for *n*_0_′′ are equivalent to the solutions of generating the right and left circularly polarized modes for *n*_0_ respectively. In addition, the directions of rotation of the circularly polarized modes can be reversed with the reversion of the direction of rotation of the electron spokes.

## Cold simulation verification

**Figure 3:**
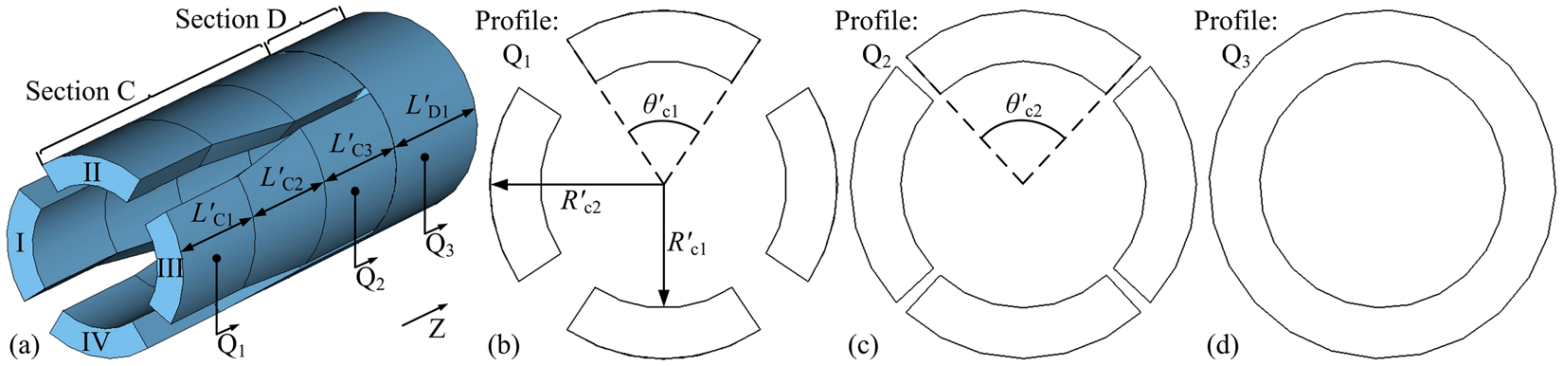
Schematic diagram of the (**a**) inner space of section C and D in an eight-cavity RM, and its transversal profiles in the (**b**) Q_1_ plane, (**c**) Q_2_ plane and (**d**) Q_3_ plane.

To verify the correctness of the theoretical analysis in section 2 in cold simulations (electromagnetic microwave simulations without simulation particles), the structure of section C and D in an eight-cavity RM (*N* = 8) as a typical example is investigated by the software CST Studio Suite^13^. The schematic diagrams of the model are shown in Fig. 3, and the structure parameters of the model are shown in Table 1. It is easy to figure out that at a frequency of 4.8 GHz for instance, only the TE_11_ mode can propagate steadily in the sectorial waveguides of section C and only the TEM, TE_11_, TE_21_, TE_31_, and TE_41_ modes can propagate steadily in the coaxial waveguide of section D, while other waveguide modes are evanescent higher order modes.

**Table 1:** Structure parameters of section C and D in an eight-cavity RM.

Parameters	*R*′_*c*1_ (mm)	*R*′_*c*2_ (mm)	*θ*′_*c*1_ (°)	*θ*′_*c*2_ (°)	*L*′_*C*1_ (mm)	*L*′_*C*2_ (mm)	*L*′_*C*3_ (mm)	*L*′_*D*1_ (mm)
Values	34.0	48.0	67.5	85.5	60.0	60.0	60.0	70.0

According to Eq. (11), for the counterclockwise rotation of electron spokes, the TE_11_ modes with the same amplitude and the initial phases of 3*n*_0_π/2, 2*n*_0_π/2, *n*_0_π/2, and 0 at 4.8 GHz are fed into sectorial waveguides I~IV of section C respectively. Then the output mode components with their corresponding proportions can be obtained from the port of section D in the cold simulations as shown in Table 2. In Table 2, *n*_0_ is 12 or a number from 1 to 7, *η* as mode conversion efficiency can be figured out by mode transmission coefficient obtained from simulation results, and the direction of rotation of the circularly polarized modes can be determined by the phase relationship between two polarized degenerate modes shown in simulations. In addition, no mode can be excited in the coaxial waveguide when *n*_0_ is 0 or 8 according to Eq. (10).

**Table 2:** Output mode components of the eight-cavity RM with their corresponding proportions in the coaxial waveguide in the cold simulations.

*n*_0_	0	1	2	3	4	5	6	7	8	12
*n*_*L*_,1 (TE_nL,1_)	/	1,1	2,1*	3,1	TEM** & 4,1*	1,1	2,1*	3,1	/	TEM** & 4,1*
*η* (TE_nL,1_)	/	71.00%	99.95%	28.93%	91.53% & 8.33%	71.00%	99.95%	28.93%	/	91.53% & 8.33%
*n*_*R*_,1 (TE_nR,1_)	/	3,1	2,1*	1,1	TEM** & 4,1*	3,1	2,1*	1,1	/	TEM** & 4,1*
*η* (TE_nR,1_)	/	28.93%	99.95%	71.01%	91.53% & 8.33%	28.93%	99.95%	71.01%	/	91.53% & 8.33%

*n_*L*_ = *n*_*R*_ indicates that the TE coaxial waveguide mode is linearly polarized, and the corresponding value of *η* is the simulation result for the linearly polarized mode.

**The TEM mode is neither linearly polarized nor circularly polarized, and the corresponding value of *η* is the simulation result for the TEM mode.

From Table 2, one can see that for the eight-cavity RM (*N* = 8) in the cold simulations when *n*_0_ = 2 or 6, the linearly polarized TE_21_ mode can be excited in the coaxial waveguide, and the solutions of generating the linearly polarized mode for *n*_0_ = 2 and 6 are equivalent; when *n*_0_ = 4 or 12, the TEM mode and the linearly polarized TE_41_ mode can be excited in the coaxial waveguide, and the solutions of generating the TEM mode and the linearly polarized mode for *n*_0_ = 4 and 12 are equivalent; when *n*_0_ = 1 or 5, the left circularly polarized TE_11_ mode and the right circularly polarized TE_31_ mode can be excited in the coaxial waveguide, and the solutions of generating the left and right circularly polarized modes for *n*_0_ = 1 and 5 are equivalent; when *n*_0_ = 3 or 7, the left circularly polarized TE_31_ mode and the right circularly polarized TE_11_ mode can be excited in the coaxial waveguide, and the solutions of generating the left and right circularly polarized modes for *n*_0_ = 3 and 7 are equivalent; comparing the two cases of *n*_0_ = 1 and 3, the solutions of generating the left and right circularly polarized modes for *n*_0_ = 1 are equivalent to the solutions of generating the right and left circularly polarized modes for *n*_0_ = 3 respectively; comparing the two cases of *n*_0_ = 1 and 7, *n*_0_ = 3 and 5, or *n*_0_ = 5 and 7, the similar conclusions also can be obtained; in addition, the directions of rotation of the circularly polarized modes can be reversed with the reversion of the direction of rotation of the electron spokes.

Obviously these cold simulation results are absolutely consistent with the theoretical analysis described in section 2. And for other cases of *n*_0_ > 8 in this model and other models of *N* ≠ 8, the theoretical analysis also can be proved correct by the cold simulations which may be not necessary to show here one by one.

## Hot simulation verification

To further confirm the correctness of the theoretical analysis in section 2, the hot simulations (electromagnetic microwave simulations with simulation particles) are run for typical RM structures. In order to obtain a pure TEM or TE_n1_ mode with slight modifications of electrical parameters or structural parameters in the hot simulations without loss of generality, a ten-cavity RM (*N* = 10) is investigated by using the CHIPIC software^14^. The initial structure of the RM model shown in Figs 1 and 2 with its structure parameters shown in Table 3 is predesigned for obtaining the TEM mode with five electron spokes. It is easy to figure out that at an operating frequency of 4.30 GHz only the TE_11_ mode can propagate steadily in the sectorial waveguides and only the TEM, TE_11_, TE_21_, and TE_31_ modes can propagate steadily in the coaxial waveguide, while other waveguide modes are evanescent higher-order modes. Since the user-specified direction of the applied axial magnetic field is along the +Z direction, the direction of rotation of electron spokes is considered counterclockwise according to the direction of ***E***_***r***_ × ***B***_***z***_, where ***E***_***r***_ is the radial component of the applied electric field and ***B***_***z***_ is the axial component of the applied magnetic field. Then, based on the theoretical analysis in section 2, with Eq. (22) the output mode components of the ten-cavity RM in the coaxial waveguide in the cold simulations can be given in Table 4.

**Table 3:** Structure parameters of the ten-cavity RM.

Parameters	*R*_*c*_ (mm)	*R*_*a*_ (mm)	*R*_*v*_ (mm)	*R*_*s*_ (mm)	*R*_*c*1_ (mm)	*R*_*c*2_ (mm)	*θ*_*v*_ (°)	*θ*_*s*_ (°)	*θ*_*c*1_ (°)
Values	11.0	18.0	30.0	32.0	34.0	48.0	18.0	18.0	54.0
Parameters	*θ*_*c*2_ (°)	*L*_*B*1_ (mm)	*L*_*B*2_ (mm)	*L*_*B*3_ (mm)	*L*_*B*4_ (mm)	*L*_*C*1_ (mm)	*L*_*C*2_ (mm)	*L*_*C*3_ (mm)	*L*_*D*1_ (mm)
Values	67.5	72.0	90.0	30.0	32.5	40.5	40.0	30.0	70.0

**Table 4:** Output mode components of the ten-cavity RM in the coaxial waveguide in the cold simulations

*n*_0_	0	1	2	3	4	5	6	7	8	9	10
*n*_*L*_,1 (TE_nL,1_)	/	1,1	2,1	3,1	(4,1)*	TEM & (5,1)*	1,1	2,1	3,1	(4,1)*	/
*n*_*R*_,1 (TE_nR,1_)	/	(4,1)*	3,1	2,1	1,1	TEM & (5,1)*	(4,1)*	3,1	2,1	1,1	/

*Indicates that the mode in the bracket is an evanescent higher order mode.

### TEM mode for *n*_0_ = 5

For obtaining the TEM mode with five electron spokes (*n*_0_ = 5), a voltage of 220 kV and an axial magnetic field of 0.4 T are applied to drive the RM on the CHIPIC software.

**Figure 4:**
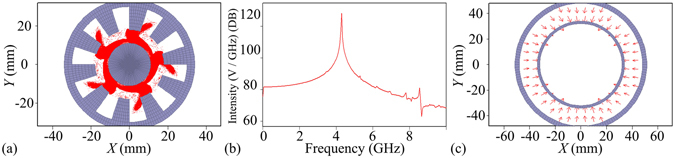
(**a**) Five electron spokes in the Rθ plane of the ten-cavity RM. (**b**) Spectrums of electric fields in dB in the output port of the coaxial waveguide from 20 to 40 ns. (**c**) TEM mode in the output port.

Figure 4(a) shows that five electron spokes in the ten-cavity RM have been completely formed in angular direction.

Figure 4(b) shows that the frequency *f* = 4.30 GHz is the dominating operating frequency since its spectral amplitude is at least 47.268 dB higher than those of other frequencies.

Figure 4(c) shows that the output mode is the TEM mode, which is consistent with the theoretical analysis and the cold simulation.

### Circularly polarized TE_11_ mode for *n*_0_ = 4

For obtaining the circularly polarized TE_11_ mode with four electron spokes (*n*_0_ = 4), a voltage of 280 kV and an axial magnetic field of 0.4 T are applied to drive the RM on the CHIPIC software, where the cathode radius *R*_*c*_ = 9 mm is used for the present case.

**Figure 5:**
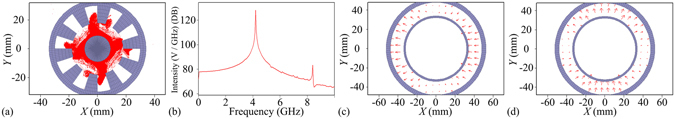
(**a**) Four electron spokes in the Rθ plane of the ten-cavity RM. (**b**) Spectrums of electric fields in dB in the output port of the coaxial waveguide from 20 to 40 ns. (**c**) TE_11_ mode in the output port at 27.949 ns. (**d**) TE_11_ mode in the output port at 29.201 ns.

Figure 5(a) shows that four electron spokes in the ten-cavity RM have been completely formed in angular direction.

Figure 5(b) shows that the frequency *f* = 4.20 GHz is the dominating operating frequency since its spectral amplitude is at least 44.894 dB higher than those of other frequencies.

Figures 5(c,d) show the output mode patterns in the output port at *t* = 27.949 ns and 29.201 ns respectively. These are both the TE_n1_ mode (*n* = 1) but with different polarization directions and can be unambiguously identified as the circularly polarized TE_11_ mode. According to the time difference of the two TE_11_ modes (∆*t* = 29.201 − 27.949 = 1.252 ns), the period of rotation of the circularly polarized TE_11_ mode (*T*_TE11_ = *n*/*f* = 1/4.2 ns), and the angle difference of the polarization, one can easily figure out that the TE_11_ mode is right circularly polarized, which is consistent with the theoretical analysis and the cold simulation.

### Circularly polarized TE_11_ mode for *n*_0_ = 6

For obtaining the circularly polarized TE_11_ mode with six electron spokes (*n*_0_ = 6), a voltage of 160 kV and an axial magnetic field of 0.4 T are applied to drive the RM on the CHIPIC software, where the cathode radius *R*_*c*_ = 13 mm is used for the present case.

**Figure 6:**
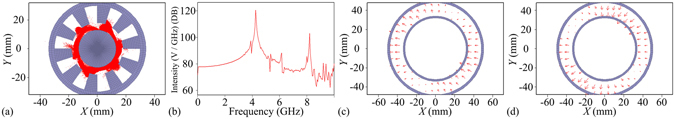
(**a**) Six electron spokes in the Rθ plane of the ten-cavity RM. (**b**) Spectrums of electric fields in dB in the output port of the coaxial waveguide from 20 to 40 ns. (**c**) TE_11_ mode in the output port at 29.323 ns. (**d**) TE_11_ mode in the output port at 30.779 ns.

Figure 6(a) shows that six electron spokes in the ten-cavity RM have been completely formed in angular direction.

Figure 6(b) shows that the frequency *f* = 4.25 GHz is the dominating operating frequency since its spectral amplitude is at least 17.6 dB higher than those of other frequencies.

Figure 6(c,d) shows the output mode patterns in the output port at *t* = 29.323 ns and 30.779 ns respectively. These patterns fall into the category of the TE_n1_ mode (*n* = 1) but with different polarization directions and they can be unambiguously identified as the circularly polarized TE_11_ mode. According to the time difference of the two TE_11_ modes (∆*t* = 30.779 − 29.323 = 1.456 ns), the period of rotation of the circularly polarized TE_11_ mode (*T*_TE11_ = *n*/*f* = 1/4.25 ns), and the angle difference of the polarization, one can easily figure out that the TE_11_ mode is left circularly polarized, which is consistent with the theoretical analysis and the cold simulation.

### Circularly polarized TE_21_ mode for *n*_0_ = 3

For obtaining the circularly polarized TE_21_ mode with three electron spokes (*n*_0_ = 3), a voltage of 320 kV and an axial magnetic field of 0.4 T are applied to drive the RM on the CHIPIC software, where the cathode radius *R*_*c*_ = 8 mm is used for the present case.

**Figure 7:**
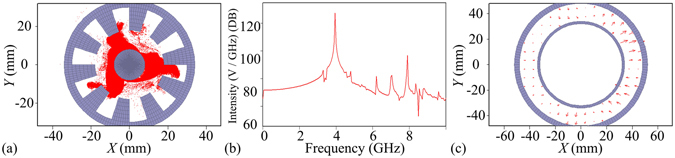
(**a**) Three electron spokes in the Rθ plane of the ten-cavity RM. (**b**) Spectrums of electric fields in dB in the output port of the coaxial waveguide from 20 to 40 ns. (**c**) Mixed modes in the output port.

Figure 7(a) shows that three electron spokes in the ten-cavity RM have been completely formed in angular direction.

Figure 7(b) shows that the frequency *f* = 4.10 GHz is the dominating operating frequency since its spectral amplitude is at least 30.614 dB higher than those of other frequencies.

However, as the theoretical analysis is given in section 2 and the output mode components of the ten-cavity RM in the coaxial waveguide in the cold simulations are shown in Table 4, both the TE_21_ and TE_31_ modes can be excited and propagate steadily in the coaxial waveguide, so that it is difficult to identify the output mode pattern shown in Fig. 7(c).

**Figure 8:**
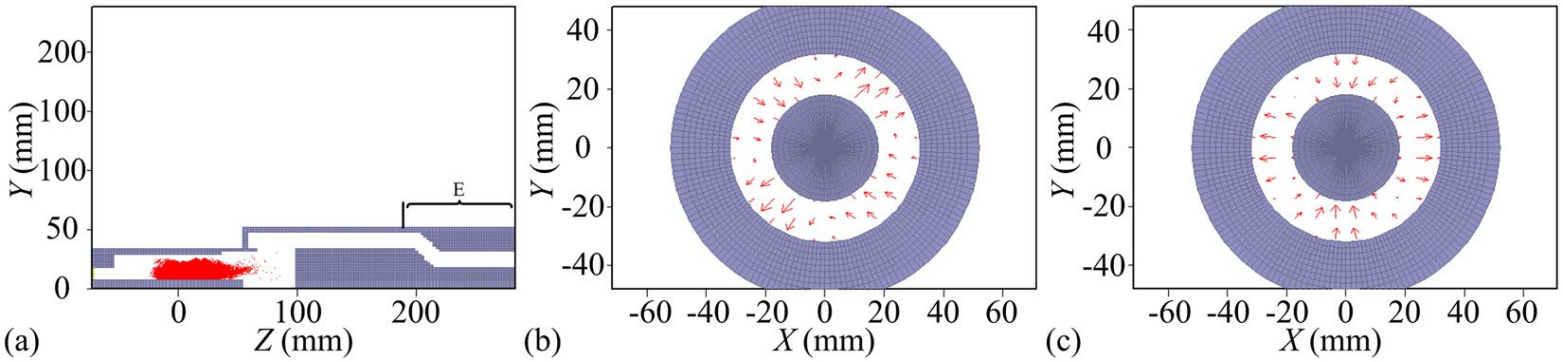
(**a**) Electron spokes in the RZ plane of the ten-cavity RM with section E replacing section D. (**b**) TE_21_ mode in the output port at 28.954 ns. (**c**) TE_21_ mode in the output port at 29.996 ns.

In order to suppress the TE_31_ mode component and obtain only the TE_21_ mode, a simple approach such as adjusting the radial dimensions of the coaxial waveguide may be feasible. As is shown in Fig. 8(a), the structure of section E consisting of a modified coaxial waveguide with the inner radius of 18.0 mm and the outer radius of 32.0 mm, and a tapered coaxial waveguide connecting the modified coaxial waveguide to the end of section C is used instead of the structure of section D.

Figure 8(b,c) show the output mode patterns in the output port of the modified RM at *t* = 28.954 ns and 29.996 ns respectively. These patterns fall into the category of the TE_n1_ mode (*n* = 2) but with different polarization directions and they can be unambiguously identified as the circularly polarized TE_21_ mode. According to the time difference of the two TE_21_ modes (∆*t* = 29.996− 28.954 = 1.042 ns), the period of rotation of the circularly polarized TE_21_ mode (*T*_TE21_ = *n*/*f* = 2/4.10 ns), and the angle difference of the polarization, one can easily figure out that the TE_21_ mode is right circularly polarized, which is consistent with the theoretical analysis and the cold simulation.

In addition, from the hot simulations above, it is worth noting that the direction of rotation of the circularly polarized modes can be easily reversed with the reversion of the direction of rotation of the electron spokes by reversing the current of the magnetic coils, while the TEM mode and the linearly polarized modes are unaffected by the direction of rotation of the electron spokes.

## Conclusion

In this paper, through theoretically analysing the physical mechanism of the radiation generation of all possible output modes of the RM with all cavity-magnetron axial extraction technique, the necessary conditions for generating the TEM mode, the linearly polarized TE_n1_ mode, and the circularly polarized TE_n1_ mode in the coaxial waveguide are obtained respectively, as well as some regular characteristics of the corresponding mode excitation, and then verified by the cold and hot simulations. Undoubtedly, these theoretical investigations provide a comprehensively and thoroughly theoretical guidance for designing this type of RM. And more importantly, they not only show that the RM has the ability of generating any a mode among the TEM mode, the linearly polarized TE_n1_ mode, and the circularly polarized TE_n1_ mode in the coaxial waveguide, but also indicate that the direction of rotation of the circularly polarized mode can be easily reversed with the reversion of the direction of rotation of the electron spokes by reversing the current of the magnetic coils. Such unique attractive properties make it possible for this type of RM to meet application requirements of various HPM modes.
